# Decoding of grasping information from neural signals recorded using peripheral intrafascicular interfaces

**DOI:** 10.1186/1743-0003-8-53

**Published:** 2011-09-05

**Authors:** Silvestro Micera, Paolo M Rossini, Jacopo Rigosa, Luca Citi, Jacopo Carpaneto, Stanisa Raspopovic, Mario Tombini, Christian Cipriani, Giovanni Assenza, Maria C Carrozza, Klaus-Peter Hoffmann, Ken Yoshida, Xavier Navarro, Paolo Dario

**Affiliations:** 1 BioRobotics Institute, Scuola Superiore Sant'Anna, Pisa (Italy; 2 Institute for Automation, Swiss Federal Institute of Technology, Zurich (Switzerland; 3 Campus Biomedico University, Rome (Italy; 4 Casa di Cura S. Raffaele, and IRCCS S. Raffaele-Pisana, Rome (Italy; 5 Fraunhofer Institute for Biomedical Engineering, St. Ingbert (Germany; 6 Indiana University-Purdue University, Indianapolis (USA; 7 Universitat Autònoma de Barcelona, and CIBERNED, Barcelona (Spain; 8 Institute of Neurology, Catholic University, Po. Gemelli, Rome, Italy

## Abstract

**Background:**

The restoration of complex hand functions by creating a novel bidirectional link between the nervous system and a dexterous hand prosthesis is currently pursued by several research groups. This connection must be fast, intuitive, with a high success rate and quite natural to allow an effective bidirectional flow of information between the user's nervous system and the smart artificial device. This goal can be achieved with several approaches and among them, the use of implantable interfaces connected with the peripheral nervous system, namely intrafascicular electrodes, is considered particularly interesting.

**Methods:**

Thin-film longitudinal intra-fascicular electrodes were implanted in the median and ulnar nerves of an amputee's stump during a four-week trial. The possibility of decoding motor commands suitable to control a dexterous hand prosthesis was investigated for the first time in this research field by implementing a spike sorting and classification algorithm.

**Results:**

The results showed that motor information (e.g., grip types and single finger movements) could be extracted with classification accuracy around 85% (for three classes plus rest) and that the user could improve his ability to govern motor commands over time as shown by the improved discrimination ability of our classification algorithm.

**Conclusions:**

These results open up new and promising possibilities for the development of a neuro-controlled hand prosthesis.

## I. Introduction

The human hand is a versatile organ that is used for grasping heavy or delicate objects and for performing highly complex manipulations on the basis of fine motor control and precise sensory feedback [1]. The restoration of these sensorimotor functions after upper limb amputation is particularly challenging. Two main components must be developed: (a) hand prostheses able to mimic the natural hand from both a biomechanical and sensory points of view [2]; (b) intimate interfaces for online bridging the user's nervous system and the external prosthesis. Several solutions are possible and are currently investigated by independent research groups using non-invasive [3-5], and invasive [6-8] approaches. For instance, intracortical signals can be used to simultaneously control reaching and one degree of freedom grasping of an artificial limb as recently shown in non-human primates [8].

In case of amputation, it is also possible to use the residual part of amputee muscles and peripheral nerve fibers to control the artificial hand. This approach can be implemented by processing electromyographic (EMG) signals recorded using either non-invasive [9] or invasive [10,11] electrodes. The transposition of residual nerves of amputees to other muscles in or near the amputation site can be also implemented [12-14]. This approach (called "Targeted Muscle Reinnervation", TMR) is probably the most advanced clinical solution currently available and it has the interesting advantage that the nerve function correlates physiologically to the motor action controlling in the prosthesis. Therefore, the control of the prosthesis is more natural and easier than with other EMG-based approaches. However, it presents two main limitations: (i) it requires a major surgical intervention and the use of a grid of surface electrodes. In this way, the problems due to the invasiveness are not totally balanced by significant advantages in terms of usability and cosmetic appearance; (ii) it is effective mainly for proximal amputation levels (i.e., close to the axilla, which are less common than transradial - adjacent to the elbow - amputations) because of the characteristics of the surgical procedure.

The use of invasive neural interfaces directly connected to the peripheral nervous system (PNS) is potentially appealing because it is able to provide an almost "physiological" condition in which efferent and afferent fibers, previously connected with the natural hand, may return to their role in controlling the prosthetic limb/hand. This is theoretically possible since a significant amount of peripheral nerve fibers, as well as spinal cord and brain connections dedicated to the control of the amputated limb, survive in time and remain available to restore a "physiological" condition also thanks to the plastic abilities of the CNS to reorganize for functional recovery [15-17].

Several invasive PNS interfaces have been developed in the past [18]. Although most of them were originally used for functional electrical stimulation (FES) in spinal cord injured persons [19-21], they can also be the key component of neuro-controlled hand prostheses. In this case, they are used to record efferent motor signals and to deliver sensory feedback [22-25].

Among invasive PNS interfaces, longitudinal intra-fascicular electrodes (LIFEs) - namely intrafascicular electrodes inserted longitudinally into the nerve [26,27] - are interesting due to the selective contact with a limited number of specific nerve fibers and the relatively low level of invasiveness required for their implantation. In fact, after appropriate control in experimental models to test their biocompatibility and efficacy [18], LIFEs have been recently used to control artificial devices [6,7,22-25] showing good results during short-term trials with human amputees. These trials showed that subjects are able to control a one-degree of freedom hand prosthesis through online processing of the efferent neural signals, and that repeatable sensory feedback may be received by stimulating afferent fibers [22,23] corresponding to the missing hand/fingers territories. Moreover, as seen in animal models [28], LIFEs allow the extraction of spikes from the signals recorded significantly increasing the decoding ability for online classification. Spike shapes associated to different fibers depend on the size and conduction speed of the fibers, on the relative distance and orientation between the fiber and the electrode, and on the inhomogeneity of the intrafascicular space. Therefore, a spike sorting approach can be used to identify the activity related to different nerve fibers, significantly increasing the decoding ability.

Starting from these encouraging results, the aim of this study was to verify whether "hand-related" actions (such as different grip types or movements of single fingers) can be decoded by processing neural motor-related signals recorded by LIFEs. This result could allow control of a dexterous hand prosthesis using the natural neural "pathway" and increase the usability of actuated artificial hands.

In particular, the following issues were addressed also as an extension of a recent study on the same case [24,25]: (i) how many degrees of freedom (or different grasping tasks) can be reliably extracted and controlled from efferent neural signals; (ii) to which extent the combined analysis of an *ensemble *of motor LIFE signals recorded when a motor command is dispatched to the amputated hand/fingers improves movement classification via the interface processor; (iii) whether there is any learning effect during the use of the interface; (iv) whether this kind of approach needs frequent re-calibrations as in the case of invasive cortical neuroprostheses.

A more general and clinically-oriented paper on the same subject, concerning the technique of LIFE insertion and LIFE signal analysis, including results on output control, sensory perception, clinical outcome and associated neuroplastic changes has already been published elsewhere [24].

## II. Materials and methods

### A. Thin-film LIFEs

**Figure 1:**
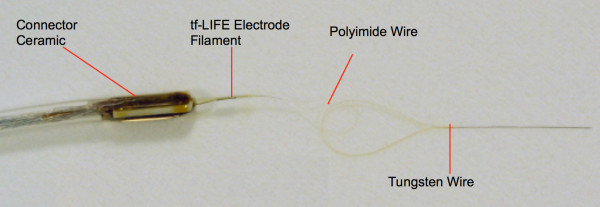
**Picture and unfolded overview of tfLIFE (17)**. Total length: 60 mm. Length without pad areas: 50 mm. Each end of the tfLIFE carries a ground electrode (GND), a reference electrode (L0, R0) and the recording sites (L1-L4, R1-R4).

A new version of LIFEs, named thin-film LIFEs (tfLIFE), was used in the experiments [29]. These electrodes were developed on a micropatterned polyimide substrate, which was chosen because of its biocompatibility, flexibility and structural properties. After microfabrication, this substrate filament (shown in Figure 1) was folded in half so that each side had four active recording sites. Therefore, tfLIFEs allow recordings at eight active sites per electrode. A tungsten needle linked to the polyimide structure was used for implanting the electrode and was removed immediately after insertion.

### B. Experimental protocol

P.P., a 26 year old male, suffered amputation of his left arm two years before the implantation due to a car accident. The surgical procedures are detailed elsewhere [24]. Briefly, following epineural micro-dissection, two tfLIFE4s (Figure 1) were inserted in the ulnar and the median nerves 45° obliquely to assure stability and to increase the probability of intercepting nerve fibers. The distal handle of the electrode was anchored to the epineurium. Four weeks later, tfLIFE4s were removed as required by the European Health Authorities. P.P. worked on the project 4-6 hours/day for 6 days/week, and did not report any complication during the following 12 months. A complete, clinical report on this case can be found elsewhere [24].

During the first three weeks of experiments P.P. was involved in several experiments addressing different biomedical and neurophysiological issues related to the efficacy of this approach (e.g., sensory feedback [24], scalp EEG recordings [30], Trancranial Magnetic Stimulation (TMS-related) neural signals [31]).

**Figure 2:**
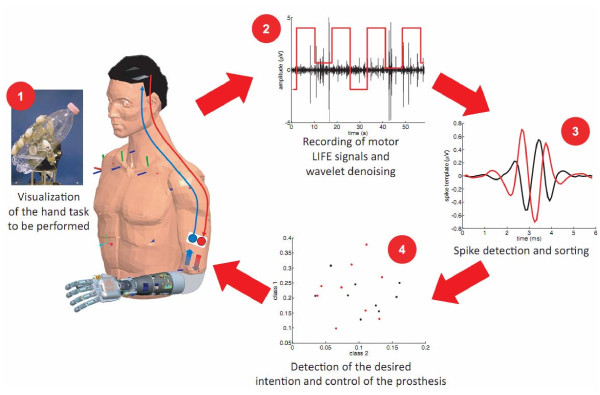
**Scheme of the algorithms used to process the LIFE motor signals and to identify the desired class of movement**. The signals recorded were denoised. This is necessary and quite useful for the commonly quite low signal-to-noise ratio of the LIFE signals [21]. The classes of spikes extracted from the LIFE signals were used as inputs for an SVM classifier able to identify the desired voluntary activity. Results about sensory feedback experiments based on electrical stimulation of afferent nerve fibers are reported in [24].

The trials on the recording of neural motor LIFE signals were carried out during the last week of experiments, after verifying an improvement in the signal-to-noise ratio of the LIFE signals. In particular, P.P. was specifically asked to separately and selectively dispatch the order to perform the following three movements: (a) palmar grasp, (b) pinch grasp, (c) flexion of the little finger. Pictures representing these tasks were randomly presented to him on a computer screen to provide a visual cue. The overall processing scheme is shown in Figure 2 and will be briefly described in the next paragraphs.

LIFE motor signals were recorded via 4-channel amplifiers (Grass QP511 Quad AC; ENG amplified: X10.000, filtered: 100 Hz- 10 kHz; 16 bit, 1 Ms/s analogue-to-digital converter).

When above a selected threshold, the neural signals recorded were used to teleoperate a dexterous hand prosthesis. The prosthetic hand used was a stand-alone version of the CyberHand prototype, already employed in several research scenarios [32]. This approach was used as a "biofeedback" to increase the confidence of the patient in this procotol.

### C. Off-line Processing algorithms of LIFE signals

A wavelet denoising technique was used to improve the signal-to-noise ratio (SNR) in the neural signals recorded. Wavelet denoising is a set of techniques used to remove noise from signals and images. The main idea is to transform the noisy data into an orthogonal time-frequency domain. In that domain, thresholding is applied to the coefficients for noise removal, and the coefficients are finally transformed back into the original domain obtaining the denoised signal [33]. A decomposition scheme based on the *translation-invariant wavelet transform *[34] was used as shown in [28]. After the denoising, the different classes of spikes were extracted. The algorithm consisted of a two-step process [28]: creation of spike templates and then comparison of spikes in the signal with the templates using some similarity indexes as the correlation coefficient or the mean square difference between a spike and a template and the power of the template. This spike-based approach was used because it already showed to allow high classification accuracy in animal experiments [28]. The characterization of the SNR was carried out according to the methodology previously described in [35].

### D. Identification of the desired motor commands

The denoised signals were then used to identify the dispatched motor commands by implementing the following procedure. For each trial, the different recording periods ('epochs') related to the movement classes (e.g., grip types and rest) were labeled. The three desired movements and the rest were considered as separated classes. The performance metrics considered was the ratios between classes correctly identified out of those presented and the leave one out validation standard method has been used.

Each epoch was an example that was used to train the classifier or to test its generalization skills. The feature vector was made of the ratios between the number of spikes matching each spike template and the total number of spikes in the epoch [28]. Therefore, the absolute spike rates were not used, but rather the relative spike rates of each waveform w.r.t. the others. This should prevent classification of the motor commands based on the "quantity of activity" and favor the use of the "quality of activity" intended in terms of different waveforms for different stimuli.

In order to infer the type of stimulus applied during a given epoch from the feature vector **F**, a classifier based on support vector machines (SVMs, [36]) was used making use of the open source library LIBSVM [37]. To allow SVMs, and other binary classifiers, to handle multiclass problems, the latter must be decomposed into several binary problems. In this work we used a one-against-one approach.

Finally, in order to assess the inter-day robustness of this approach the classifier was trained by using all the features extracted for the first day of recordings during the last week of experiments and used without any further change for the next days. This was done to understand whether this approach might be used without any need for recalibration.

## III. Results

**Figure 3:**
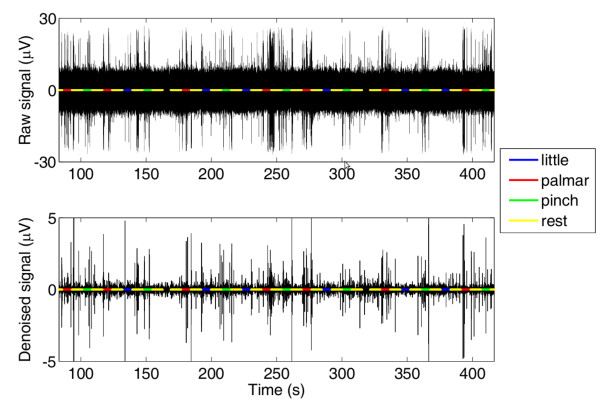
**The raw (top panel) and the denoised (bottom panel) LIFE motor signals**. Superimposed with the signal (black line) are shown signals, which represent the different tasks the subject was asked to perform (rest = yellow line; flexion of the little finger = blue line; palmar grasp = red line; pinch grasp = green line).

An example of a LIFE motor signal before and after the wavelet denoising is given in Figure 3.

**Figure 4:**
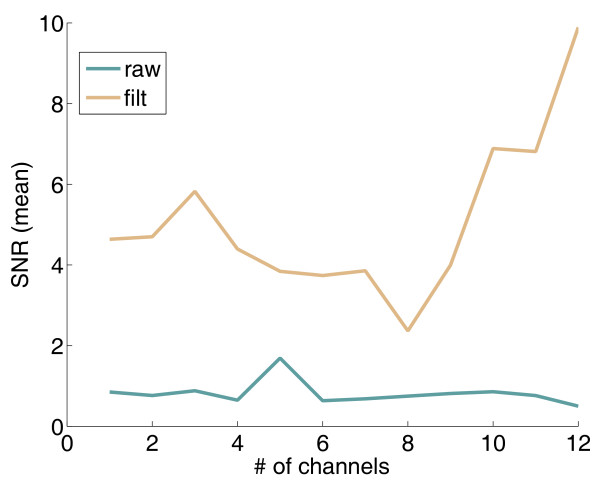
**SNR before and after the denoising for the different channels**.

The SNRs for the different channels before and after the denoising are given in Figure 4. The quality of the recording after denoising can be considered good similarly to the considerations done in [35].

**Figure 5:**
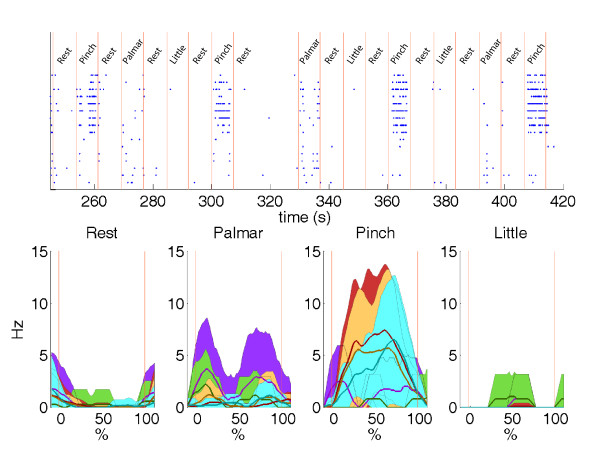
**Raster plots and spike rates**. Top panel: raster plots for different classes of spikes during the selection of each type of movements by the subject. Bottom panel: corresponding spike rates for the corresponding classes windows (mean = larger lines; mean ± SD = smaller lines and colourful areas; areas are colored when not overlapped). Window duration for each class has been normalized (from 0 to 100%). The red vertical lines delimit the timing of the classes triggering in both top and bottom panels. Only windows with the fixed width of the controlled experiment have been used.

In Figure 5, the raster plots for different classes of spikes (different colors) for the different movements are provided. A different modulation for different tasks can be seen showing that the neural signals recorded and, in particular, the classes of spikes extracted can be considered related to different motor commands. It is also interesting that we have a double-peak spike rate especially for the palmar grasp. This could allow in the future the use of the two peaks for the pre-shaping (opening) and closing of the prosthetic device.

In Table 1, the best performance achieved with the best combination of tfLIFE channels is shown for the decoding of rest plus one, two, or three movement classes are shown in the Table 2. In most cases, a combination of features coming from all the three tfLIFE channels is required to obtain the best recognition ratio (rr). Most of the channels we used were the "expected ones" from a neurophysiological viewpoint but some unexpected channels were also used. This could be due to some inability of the user to deliver the natural ("correct") neurophysiological commands.

**Table 1:** Performance of the classifier (recognition ratio) with re-training of the classification algorithm

Task(s) identified	rr	M1 channel #				M2 channel #				U channel #			
	(%)	1	2	3	4	5	6	7	8	9	10	11	12
rest vs little	93	**--**	**--**	**--**	**--**	**X**	**--**	**--**	**--**	**X**	**X**	**--**	**X**

rest vs palmar	95	**X**	**--**	**--**	**--**	**--**	**X**	**--**	**--**	**X**	**--**	**--**	**--**

rest vs pinch	100	**--**	**X**	**--**	**X**	**X**	**--**	**--**	**X**	**--**	**X**	**--**	**--**

rest vs little vs palmar	92	**--**	**X**	**--**	**--**	**X**	**--**	**X**	**--**	**--**	**X**	**--**	**X**

rest vs little vs pinch	90	**--**	**X**	**--**	**X**	**--**	**X**	**--**	**X**	**--**	**--**	**X**	**--**

rest vs pinch vs palmar	87	**--**	**--**	**X**	**--**	**X**	**--**	**--**	**--**	**--**	**--**	**--**	**X**

rest vs little vs pinch vs palmar	85	**--**	**X**	**--**	**--**	**X**	**--**	**X**	**--**	**X**	**X**	**--**	**--**

rest vs activity	87	**--**	**--**	**--**	**X**	**X**	**X**	**X**	**X**	**--**	**X**	**X**	**X**

little vs pinch vs palmar		**--**	**X**	**X**	**--**	**--**	**--**	**--**	**X**	**--**	**X**	**--**	**X**

The parameters of the classifier were selected for each day using data recorded in that specific day for the training. For each classification, the max performance (rr) and the best combination of channels used are given. rr = recognition ratio of the SVM classifier; M1 an M2 indicate the two tfLIFE implanted in the median nerve whereas U indicates the tfLIFE implanted in the ulnar nerve.

**Table 2:** Performance of the classifier (recognition ratio) without and with re-training of the classification algorithm

Grip type(s) identified and controlled (plus rest)	Little, pinch, palmar	Little, palmar	Little, pinch	Palmar, pinch	Little	Palmar	Pinch
**No Retraining**	0.72	0.86	0.84	0.8	0.9	0.76	0.9

**Retraining**	0.85	0.92	0.9	0.87	0.93	0.95	1

In case of "re-training" the parameters of the classifier were selected for each day using data recorded in that specific day for the training. In case of "no training", the definition of the parameters happened only for the first day.

In Table 2, the analysis of the advantages connected with the daily retraining of the classifier is shown. In particular, the first row shows the best results achieved when the classifier was trained only during the first day while the second row provides performance when the algorithm was re-trained everyday.

**Figure 6:**
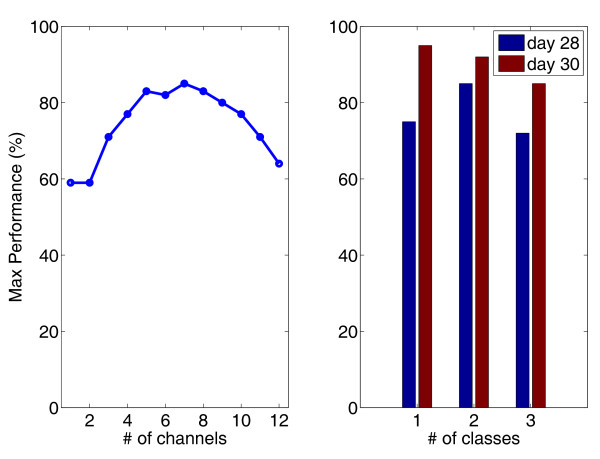
**Classification performance**. Right Panel: Improvement of the classification performance for one, two, or three classes during two training days. Left Panel: Maximum performances are shown as a function of the number of channels simultaneously acquired during the motor commands delivered to the missing limb.

The improvement of performance could be due not only to a limit in the robustness of the approach (i.e., the time-variance of the LIFE signals recorded during the different days) but also to the learning ability shown by the user. In fact, Figure 6 (right panel) shows the classification performance achieved for different classes during two days of recording. The learning process of the subject is a distinctive feature of this approach.

In order to understand the importance of using multi-channel electrodes for neural recordings, classification performance was calculated and compared using different recording channels. The results shown in Figure 6 (left panel) indicate that increasing the number of channels used for the classification enhances the performance because of the increased amount of information. Above a certain limit, performance starts degrading again, given the classic overfitting issues of classifiers (e.g., there is an excessive number of parameters to be optimized).

## IV Discussion

Among the different approaches for restoring the link between the external world and the nervous system, the use of implantable intrafascicular PNS interfaces is of great potential, as confirmed in previous experiments in humans [6,7]. The evolution of peripherally-controlled hand prostheses seems to go towards a more natural and ideal approach which is the general evolution of artificial devices. In fact, differently from the processing of surface EMG signals, which prevents the use of more natural neural "channels", the TMR approach is more natural because of the reconnection of peripheral nerves previously linked to the amputated limb but it still relies on surface EMG signals. To this respect, the use of implantable interfaces into the PNS could allow the restoration of the pre-existing neural connection completing this evolution.

The aim of our study was to increase the understanding of the basic mechanisms, as well as of the possibilities and limits of this approach, by processing the motor signals recorded the nerve fibers as final output to the target muscles (via tfLIFE signals analysis).

Two main assumptions were tested: (1) the extraction of spike templates can allow good decoding performance; (2) it is possible to extract more than a simple one degree of freedom control signal (as previously done by other researchers [7]). In particular, more complex information related to grip types can be decoded from motor signals recorded using tfLIFEs.

Concerning the first assumption we moved from previous knowledge [28] that an algorithm based on spike sorting can improve the decoding performance with respect to other more classical approaches based on the extraction of power-related information from the signal. In particular, the spike-sorted data can effectively increase the resolution of the information that can be extracted from a single electrode. This could improve the decoding performance and to some extent reduce invasiveness since fewer electrodes would be required to obtain the needed number of information channels.

The idea of decoding complex high-level information (i.e., grip types) represents the main novelty of this study. It is a disruptive approach for decoding and potentially quite challenging because of the characteristics of the signals to process. In fact, signals recorded from the peripheral nerves are related to low-level information (i.e., the commands to control muscular contraction). Therefore, this "high-level" decoding could be considered similar to understand a global picture (the grip type) with only a few pieces (related to specific muscle commands) of it. This strategy would allow an easier, more efficacious and faster (namely online) control of a dexterous hand prosthesis. In fact, this could increase the number of motor commands which could be dispatched to the hand without a very precise decoding necessary for example for the simultaneous control of the kinematics of several joints.

The results of this study, in terms of rate of correct classification of motor tasks, show that the two assumptions were correct, based on good performance achievement and a state control algorithm implementation. This control approach is commonly used in the EMG-based control of hand prostheses [38] and has been recently implemented in cortical neuroprostheses [39]. For the first time, three different hand movements were identified with good performance. This could allow the full usability of dexterous prostheses by the user who simply dispatches motor commands for hand movements as he normally did before amputation. Moreover, it could be possible to use the double-peak distribution shown for the palmar grasp (Figure 5) to separately control the pre-shaping (opening) and closing of the hand prosthesis.

Of great interest was that our amputee subject was able to improve his performance during the consecutive trials. This was particularly evident during the experiments by looking at the SNR and was also confirmed by the classification results. However, this was achieved only for two consecutive days and this learning ability has to be confirmed during longer tests.

The performance also improved by using several intrafascicular recording channels. This is due to the intrinsic blindness of the implantation procedure: a large number of channels can increase the probability of picking up enough signals to reach good classification levels and to maintain such a rate of appropriate classification stable in time. In fact, an important characteristic of our approach was the robustness of classification during the trials. This kind of interface seems to be quite stable and makes the daily re-calibration of the algorithm - which affects other implantable neuroprostheses - unnecessary. This is very important and could represent a significant advantage during daily activities carried out outside a controlled laboratory environment.

Unfortunately, due to the time limitations of our experiments it was not possible to increase the number of grip types that the subject was asked to perform. However, results clearly indicate that multiple movements can be governed, probably more than the three used in the present study. Moreover, it is also possible to combine the state control approach presented in this paper to select different grasping tasks together with a proportional control (already achieved in [6,7]). This will allow both the modulation of force during grasping and the simultaneous control of hand and elbow functions.

In the near future, the possibility of verifying these findings during chronic experiments with an implanted hand prosthesis for daily activity uses, will be investigated. The analysis of the performance of the LIFE electrodes during long-term implants is very important to understand the potentials and shortcomings of this technology in terms of chronic usability, stability, etc. Moreover, particular attention will be given to characterize long-term changes in the cortical activation and new approaches will be explored for characterizing the long-term usability and for improving the efficacy of intrafascicular electrodes. For example, as previously shown for the central nervous system [40], actuation of the active sites of the electrodes [41,42] may help to increase the SNR and selectivity both for stimulation and recording.

## Competing interests

CC hold shares in Prensilia Srl, the company that manufactures robotic hands as the one used in this work, under the license from Scuola Superiore Sant'Anna.

## Authors' contributions

SM and PMR contributed to all the stages of this work (i.e., design of the protocol, following all the experiments, data interpretation and writing). JC, LC, SR, and JR developed decoding algorithms for signal processing and classification. MT and GA contributed to the in-vivo experiments and all the clinical training. CC and MCC designed the algorithms for the control of the hand prosthesis. PD, XN, and KY contributed to the design of the protocol. KPH designed tf-LIFEs. All authors read and approved the final manuscript.
